# Comparing the quality of life of endometriotic patients’ before and after treatment with normal and infertile patients based on the EHP30 questionnaire

**DOI:** 10.1186/s12905-022-02052-x

**Published:** 2022-12-28

**Authors:** Tahereh Poordast, Saeed Alborzi, Elham Askary, Malihe Sousani Tavabe, Fatemeh Sadat Najib, Alireza Salehi, Hossein Molavi Vardanjani, Neda Haghighat, Kimia Leilami

**Affiliations:** 1 Department of Obstetrics and Gynecology, School of Medicine, Infertility Research Center, Shiraz University Of Medical Sciences, Shiraz, IR Iran; 2 Department of Obstetrics and Gynecology, School of Medicine, Laparoscopy Research Center, Shiraz University of Medical Sciences, Shiraz, IR Iran; 3 Department of Obstetrics and Gynecology, School of Medicine, Maternal-Fetal Medicine Research Center, Shiraz University of Medical Sciences, Shiraz, IR Iran; 4 Research Center for Traditional Medicine and History of Medicine, Shiraz University of Medical Sciences, Shiraz, IR Iran; 5 Department of MPH, Shiraz Medical School, Shiraz University of Medical Sciences, Shiraz, IR Iran; 6 Laparoscopy Research Center, Shiraz University of Medical Sciences, Shiraz, IR Iran

**Keywords:** Endometriosis, Radical surgery, Medical treatment, Quality of life

## Abstract

**Objective:**

This study aimed to determine the quality of life (QOL), in patients with endometriosis ± infertility (B and C groups) and compare those to healthy women, and also infertile groups without endometriosis as a control groups (A and D), considering the fact that endometriosis and infertility reduces the quality of life in patients.

**Methods:**

The present prospective comparative study was carried out between January 2018 and September 2020. A total of 400 women were included (100 women in each group). The participants filled in a validated questionnaire of quality of life, Endometriosis Health Profile-30 (EHP-30), and a visual analog scale of pain used, at the first visit, and 3 months after the medical or surgical treatment in the endometriosis group without infertility, additionally.

**Results:**

The majority of the patients were married, categorized in the middle-class of socio-economic state and housewives. They were of Persian descent. BMI was high in the infertile groups; however, the time of infertility was not different between the two groups of B and C (*P* = 0.054). The mean score of QOL was significantly lower in B, C, and D groups in comparison to the healthy women as the control group (A) (*P* < 0.001). Moreover, the infertile group (B), in comparison to endometriosis ± infertility groups (C and D), had the lowest mean score of QOL (*P* < 0.001). In each group, those who were older and had better educational level reported a better quality of life than other participants in that group. Social support plays a very important role in reducing the endometriosis related pain symptoms both before and after treatment. Three months after the treatment of endometriosis (D), a significant improvement was observed in all the aspects of QOL-related endometriosis. Nonetheless, the improvement of the quality of life in the surgical group was significantly higher than that in the medical treatment. The mean visual analog score of pain decreased from 62.22 ± 22.78, to 5.15 ± 2.73 following the surgical treatment (*P* < 0.001).

**Conclusion:**

The lowest quality of life belonged to the infertile group, followed by the endometriosis group. The quality of life of the endometriosis group improved after the treatment. Thus, endometriotic patients’ treatment in terms of improvement of quality of life should be considered by all professional health care teams.

## Introduction

Endometriosis is characterized by the expansion of functional endometrial tissue beyond the uterine space [1]. According to previous studies, approximately 0.5–10% of women in the reproductive age have endometriosis [2, 3], about one-third of whom are infertile [2].

Women with endometriosis may be asymptomatic or report symptoms of dysmenorrhea, deep dyspareunia, chronic pelvic pain, urinary pain or intestinal pain, and infertility, all of which can dramatically influence a person’s life physically and mentally [4–7].

Pain symptoms in endometriosis may not related to the severity of disease. Some authors consider psychological and emotional factors of patients to be effective on the patient’s perception of pain intensity. In addition to psychological problems such as somatization, anxiety and mood disorders in endometriosis patients, pain symptoms can disable these patients by affecting their social relationships, mental health and sexual life [8, 9]. Among these cases, extrapelvic endometriosis, which is associated with delayed diagnosis and high morbidities, should not be ignored [10].

Several articles have investigated the impact of endometriosis on the quality of life of patients, but due to the great heterogeneity in the sample size and their research methods, we have not been able to obtain high evidence conclusions in this field [11]. According to murrizo et al., systematic review treatment does not necessarily ensure the complete remission of its symptoms, but may only contribute to improving the patients’ QOL and decrease pain score [12]. Therapeutic options include no treatment, medical, surgical, or combination therapy, which is highly desirable to be based on the needs of the patient individually [13]. However, the adverse effect of medical and surgical treatments on the mental status of the endometriotic patients cannot be ignored [14]. Hormonal treatments can cause mood and sexual arousal disturbances in endometriosis patients, and surgical treatments, especially in cases of deep rectal endometriosis lesion or ureteral involvement, which often associated with parametrial involvement, can lead to some neurological complications [15, 16]. It has been observed that symptoms such as voiding dysfunction and rectal inhibitory reflex do not improve even after nerve sparing surgeries in endometriotic patients [17]. Therefore, it is necessary to examine the quality of life in endometriosis patients with and without infertility and compare the effects of surgical or medical treatment on the quality of life of these patients.

According to the World Endometriosis Societies (WES), medical treatment is a cost-effective, easy, and accessible method for these patients, and some drugs, such as oral contraceptive (OCP) pill, non-steroidal anti-inflammatory drugs (NSAID), and even progestin, can be used in combination [18]. However, on a number of occasions, for instance in case of intolerable pain symptoms, an advanced stage of the disease or unresponsiveness to medical treatment, surgical intervention was indicated [19].

There are different validated questionnaires for evaluating QOL in endometriotic patients; however, the EHP-30 is currently known as a disease-specific valid, reliable, and responsive questionnaire measuring health-related QOL in women with endometriosis [20].

This study aimed to evaluate the quality of life in endometriotic women and draw a comparison between women suffering from infertility and normal population. In the endometriosis group, we re-examined the effect of infertility on the quality of life of these patients and finally, we evaluated the effect of medical or surgical treatment on improving the quality of life in women with endometriosis and compared them to the other groups. To this end, the endometriotic patients without infertility filled out the quality of life assessment for (EHP 30) twice, once before the treatment and once after that.

## Material and methods

### Design and setting of the study

The present prospective study was conducted on premenopausal women between January 2018 and December 2020 at the Gynecologic Clinic of the Shiraz University of Medical Sciences, Shiraz, Iran.

This research followed the protocol of the Declaration of Helsinki and was approved by the Ethics Committee of Shiraz University of Medical Sciences, Shiraz, Iran (IR.SUMS.MED.REC.1399.272). Informed consent was obtained from the patients at the time of inclusion.

### Characteristics of the participants and the setting

Herein, we recruited a total of 400 premenopausal women aged 18 to 50 years old, which had attended the Gynecologic Clinic of the Shiraz University of Medical Sciences, Shiraz, Iran, from January 2018 to December 2020. They were recruited after a specific consultation in the outpatient clinic. Convenience sampling was applied since the authors had direct access to the gynecologic outpatient clinic; accordingly, the minimum sample size of 400 participants was reached. Our assumptions for sample size calculation were as follows: a type I error of below 0.05; a statistical power of 80%; a minimum mean difference of five; a mean of quality of life score of 20 in the control group; a response rate of 85% in the follow-up. Accordingly, the minimum sample size was calculated at 102 patients in each group.

### Data collection

The sampling was continued until reaching 100 patients in each group.

The endometriotic patients diagnosed with infertility were referred to infertility clinics, and those who did not intend to be pregnant were considered for medical or surgical therapy, based on the characteristics of each patient.

### Selection criteria

The inclusion criteria were as follows: endometriotic women, infertile women, those who had agreed to participate in the study as healthy subjects, and those who had signed the consent in addition to post-consent forms in the endometriosis group and received treatment during the follow-up period.

The exclusion criteria were the age of below 18 years, male factor infertility, women with cognitive impairment, and those with other chronic diseases, such as neoplasia and rheumatological diseases, which could impact the QOL.

### Data collection and measures

#### Diagnosis of endometriosis and infertility

Preoperative diagnosis of endometriosis was based on a patient’s clinical symptoms, which were then confirmed via trans-vaginal ultrasound. Infertility was considered in patients aged < 30 years old with the failure to conceive after a year of unprotected intercourse or those aged > 35 with the failure to conceive after 6 months of unprotected intercourse.

### Quality of life and pain measurements

The participants were asked to fill in a validated questionnaire of quality of life [Endometriosis Health Profile-30 (EHP-30)] and a visual analog scale (VAS) of pain at the first visit. EHP-30 questioner, which was developed by G Jones et al. [18], is a reliable and disease-specific instrument for evaluating the health-related quality of life of women with endometriosis. It consists of 30 questions in two parts, five items (including pain, control and powerlessness, emotional well-being, lack of social support, and self-image) from the core questionnaire and six items (including work, intercourse, and worries about infertility, treatment, and relationship with children and medical professionals) from the modular questionnaire [19]. Nojomi et al. evaluated the reliability and validity of this questionnaire in Iran [20]. The score given to each item ranges between zero and four (never = 0, rarely = 1, sometimes = 2, often = 3, always = 4, and not relevant if not applicable). Each scale is calculated based on the total of raw scores of each item in the scale divided by the maximum possible raw scores of all the items in the scale multiplied by 100. The scores of the scales are standardized on a range of zero to 100. Lower scores indicate a more favorable status.

The visual analog scale (VAS) of pain comprises a horizontal line of 10 cm in length anchored by the verbal descriptors of no pain (score of zero) and worst imaginable pain (score of 10) [20]. The patients were asked to place a line perpendicular to the VAS line at the point that represented their pain intensity. According to the VAS, the scores for endometriosis-related pain for dyspareunia, dysmenorrhea, and non-menstrual pelvic pain were assessed. The baseline characteristics of the patients (age and parity), the presence or absence of major symptoms associated with endometriosis (dysmenorrhea, dyspareunia, dyschezia, or chronic pelvic pain (CPP)), and the history of previous surgery for endometriosis were noted down at the preoperative visit.

At phase 2 of the study, the patients with endometriosis without infertility (group D) were evaluated and asked to fill in EHP-30 and VAS of pain 3 months after the surgical (*n* = 54) or medical (*n* = 48) treatments.

### Statistical analysis

Data were prepared applying appropriate statistical approaches. The data concerning the continuous variables are presented as mean ± SD. Paired-samples t-test and independent-samples t-test were respectively used for univariate comparison of the within- (before-after) and between-group values of QOL and pain score. The percentage of changes in QOL and pain scores was also calculated as month 3 values - baseline values) / baseline values × 100. Multivariable ordinary least squared (OLS) regression was utilized to investigate the association between different independent variables and the QOL. The variable selection for multivariable modeling was done based on a univariate *P*-value of below 0.3. The final multivariable model was fitted using a backward elimination technique. Adjusted risk deference (RD; standardized regression coefficient) and their 95% CI were estimated. A two-tailed *P*-value of ≤0.05 was considered to be statistically significant. Statistical analysis was performed via SPSS 22 for Windows (SPSS, Inc., Chicago, IL, USA).

## Results

After excluding one patient from group B and three patients from group C due to unwillingness to further cooperation, the study was conducted with 100 patients in group A, 99 in group B, 97 in group C, and 102 patients in group D. The demographic data are summarized in Table 1. The majority of the subjects in the four groups were married. Regarding job status in all groups, they were mostly housewives. The ethnicity of around 68% (271) of the participants was Persian, and 64.3% (256) of the subjects had the medium socio-economic status. There were differences in terms of body mass index (BMI) among the groups, and considering a BMI of 25 as the boundary of obesity, 55.6% of the infertile women without endometriosis passed this limit while in the other three groups, normal BMI was seen more frequently.

**Table 1:** Demographic data of all the four studied groups

Number of the patients in each group	Infertility without endometriosis ***N*** = 99	Endometriosis with infertility ***N*** = 97	Endometriosis without infertility ***N*** = 102	Normal population ***N*** = 100	***P*** value
Age					
≤ 30	38 (38.4)	36 (37.1)	29 (28.4)	50 (50)	0 < .001
30-40	57 (57.6)	51 (52.6)	44 (43.1)	32 (32)	
> 40	4 (4)	10 (10.3)	29 (28.4)	18 (18)	
BMI					
< 25	44 (44.4)	64 (66)	64 (62.7)	91 (91)	0 < .001
≥ 25	55 (55.6)	33 (34)	38 (37.3)	9 (9)	
Marital Status					
Single	0	1(1.0)	35 (29.4)	27 (27)	0 < .001
Married	99 (100)	96 (99)	72 (7.06)	73 (73)	
Education					
Low	43(43.4)	39 (40.2)	30 (29.4)	12 (12)	0 < .001
Medium	33(33.3)	30 (30.9)	38 (37.3)	39 (39)	
High	23(23.2)	28 (28.9)	34 (33.3)	49 (49)	
Job					
Housewife	94(94.9)	87 (89.7)	72(70.6)	67 (67)	0 < .001
Employee	5(5.1)	7 (7.2)	16 (15.7)	15 (15)	
Other	0	3 (3.1)	14 (13.7)	18 (18)	
Socio-economic status					
Low	32 (32.3)	54 (55.7)	35 (34.3)	21 (21)	.001
Medium	67 (67.7)	43 (44.3)	67 (65.7)	79(79)	
Ethnicity					
Persian	65 (65.7)	65 (67)	75 (73.5)	66(66)	.20
Others	34 (34.3)	32(33)	27(26.5)	34(34)	
Residency					
Center of province	57(57.6)	41(42.3)	58 (56.9)	96(96)	.053
Others	42(42.4)	56(57.7)	44 (43.1)	4(4)	
Previous live birth					
No	99(100)	97(100)	9(8.8)	–	0 < .001
Yes	0(0)	0(0)	63(61.8)	–	
Single woman	0(0)	0(0)	30 (29.4)	–	
Previous surgery for endometriosis					
No	99 (100)	89 (91.8)	93(91.2)	–	0 < .001
Yes	0 (0)	8 (8.2)	9 (8.8)	–	
Menstrual cycle					
Regular	70 (70.7)	84 (86.6)	67 (65.7)	–	.002
Irregular	29(29.3)	13(13.4)	35 (34.3)	–	
First menarche age	12.97 ± 1.79	12.68 ± 1.52	12.93 ± 1.54	–	.40
Duration years of Infertility	7.07 ± 4.79	5.67 ± 5.3	–	–	.054

*BMI* body mass index [21]

All the results are presented as number (percentage) or mean ± SD

P: *P*-value refers to comparisons between the groups (Kruskal-Wallis test or ANOVA as appropriated)

According to the clinical data of the participants, 70.7% of the infertile women without endometriosis had a regular menstrual cycle, and the mean age of onset of menstruation in this group was 12.97 ± 1.79. Among the infertile cases with endometriosis, 86.6% experienced regular menstrual cycles and had a lower onset age of 12.68 ± 1.52. Finally, in the endometriosis group without infertility, the percentage of women with the regular menstrual cycle was 65.7% and the mean onset age in this group was 12.93 ± 1.54. Additionally, 94.2% (281) of these three groups had no previous surgery for endometriosis, 74.1% (221) of whom had no history of major medical diseases.

The QOL according to the results of the EHP30 questionnaire in the study groups.

**Fig. 1:**
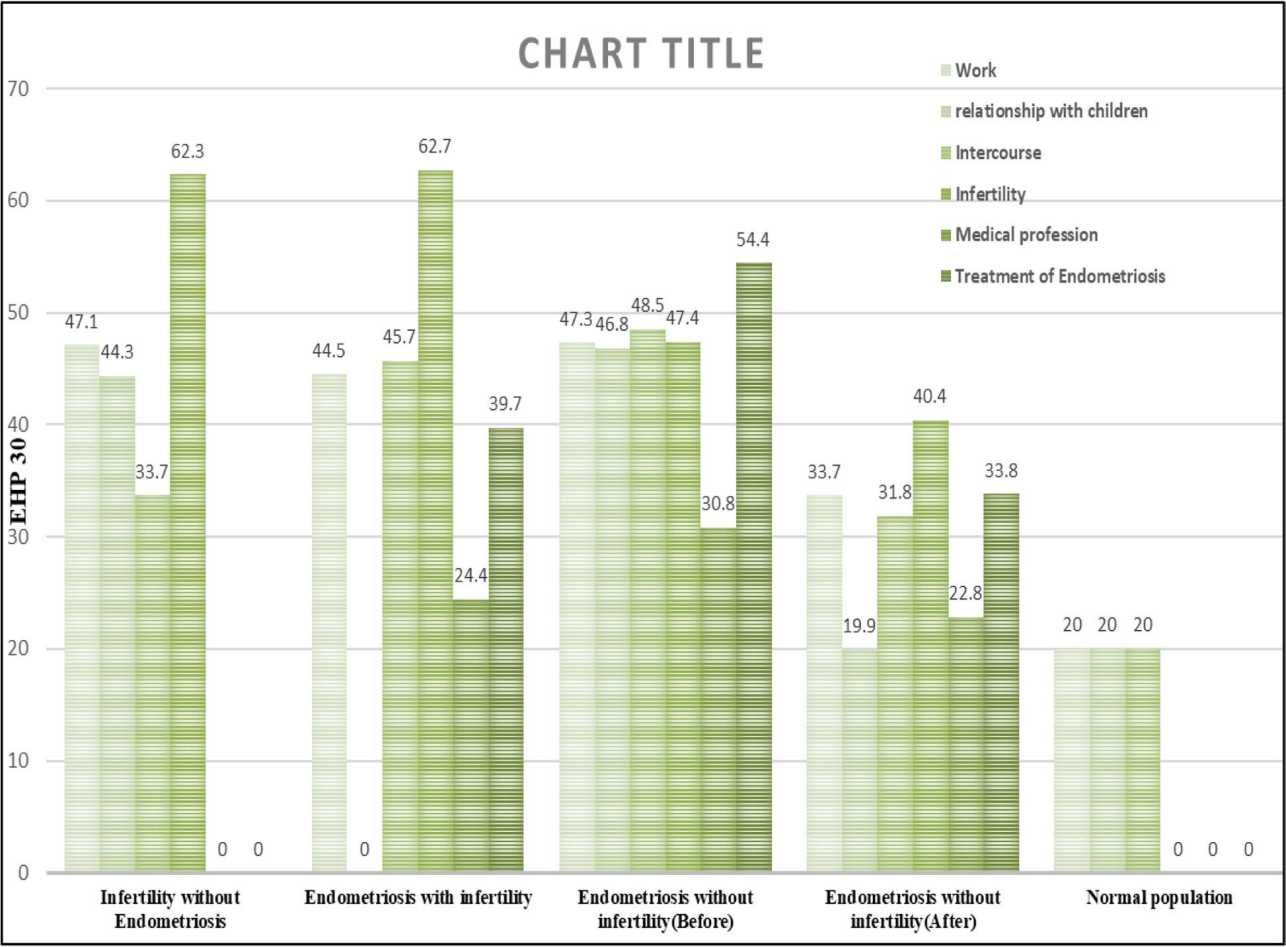
Comparing the quality of life via EHP30 questionnaire in all the patients

To investigate the quality of life according to the results of the EHP30 questionnaire, the mean score ± SD of each domain was measured; the results are reported in Tables 2 and 3. After comparing the total average of each group, significant differences were observed between the infertile women without endometriosis, infertile ones with endometriosis, and the endometriotic group without infertility (*P* < .001). However, there was no significant difference between the two infertile with endometriosis and fertile with endometriosis groups, who had almost the same average quality of life. Ultimately, the results of the comparison of the mean quality of life among all the three groups of infertile without endometriosis, infertile with endometriosis, and fertile with endometriosis with the normal group were quite significant (*P* < 0.001) (Fig. 1).

**Table 2:** Quality of life via EHP30 questionnaire in all the four studied groups

Variable	infertility without Endometriosis *N* = 99	Endometriosis with infertility *N* = 97	Endometriosis without infertility *N* = 102	normal population *N* = 100	*P* value
Pain	32.49 ± 13.83 ^b^	53.90 ± 22.61	58.61 ± 20.94	20.0 ± 0.0 ^a^	< 0.001
Control and powerlessness	55.63 ± 22.39	62.61 ± 23.42	62.51 ± 23.08	20.0 ± 0.0 ^a^	< 0.001
Emotional well-being	56.56 ± 20.6	62.57 ± 23.23	59.77 ± 21.77	20.0 ± 0.0 ^a^	< 0.001
Social support	60.10 ± 21.09	57.26 ± 26.21	57.54 ± 22.91	20.0 ± 0.0 ^a^	< 0.001
Self-image	45.52 ± 21.42	48.31 ± 22.32	45.29 ± 23.90	20.0 ± 0.0 ^a^	< 0.001
Total	34.943 ± 11.59 ^a^	57.27 ± 19.47	58.15 ± 18.89	20.0 ± 0.0 ^a^	< 0.001
Work	47.06 ± 19.7	44.44 ± 18.5	47.26 ± 21.5	20.0 ± 0.0^a^	< 0.001
relationship with children	44.28 ± 21.5^b^	–	46.83 ± 29.96	20.0 ± 0.0^a^	< 0.001
Intercourse [22]	33.7 ± 17.2 ^b^	45.68 ± 26.43	48.52 ± 23.19	20.0 ± 0.0 ^a^	< 0.001
Infertility	62.3 ± 21.67	62.66 ± 22.04	47.36 ± 25.22 ^a^		< 0.001
Medical profession	–	24.42 ± 9.88	30.78 ± 14.24		.003
Treatment of Endometriosis	–	39.74 ± 25.96	54.43 ± 24.33		.01

All the results are presented as the mean ± SD

P: *P*-value refers to comparisons between the groups (Kruskal-Wallis test or ANOVA as appropriated)

All *P*-values for pair wise between-group differences were obtained by post hoc bon ferroni test; only statistically significant results are shown

_a_*P* < 0.001 versus control

_b_*P* < 0.05 versus control

**Table 3:** Endometriosis-related pain and self-rate assessment via VAS score in three groups

Variable	infertility without Endometriosis *N* = 99	Endometriosis with infertility *N* = 97	Endometriosis without infertility *N* = 102
Noncyclic pain	3 ± 0.82	6.39 ± 2.32	7 ± 2.18
Dysmenorrhea	4.83 ± 1.66	7.8 ± 2.33	7.86 ± 2.14
Dyspareunia	3.5 ± 1	6.44 ± 2.56	6.36 ± 2.47
GI symptom at time of mensturation period	3 ± 0	6.32 ± 2.26	6.87 ± 2.53
Urinary symptom at time of mensturation period	6	6.29 ± 2.38	6.45 ± 2.23
Total	4.06 ± 0.95	6.65 ± 2.40	6.91 ± 2.47

All the results are presented as the mean ± SD

Furthermore, to evaluate the effect of the variables of different groups, namely age and level of education, on the quality of life based on EHP30 questionnaire, a regression analysis was performed, the results of which are summarized in Table 4. The decrease in the quality of life in each group was reported as a coefficient of values of the reference group belonging to the same group. In each group, those who were older and had better educational level reported a better quality of life than other participants in that group. According to this table, the quality of life, in general, significantly improves in line with age and education level, particularly in the endometriosis and infertility groups and those without infertility, the quality of life was 35 times and 37 times lower than that in the normal reference group, respectively.

**Table 4:** The effect of educational level and age on quality of life according to EHP30 questionnaire

Independent variables	B ± SE^a^	95% CI^a^	***P***-value
**Educational level**			
**Less than diploma**	**4.5 ± 2.0**	**(.5,8.4)**	**.03**
**Diploma**	**3.75 ± 1.8**	**(0.1,7.3)**	**.04**
**Bachelor and higher**	**Reference.**		
**Group**			
**Infertility without Endometriosis**	**12.33 ± 2.3**	**(7.6,17.0)**	**0 < .001**
**Endometriosis with infertility**	**35.65 ± 2.2**	**(31.2,40.0)**	**0 < .001**
**Endometriosis without infertility**	**37.63 ± 2.1**	**(33.4,41.7)**	**0 < .001**
**Normal population**	**Reference.**		
**Age**			
**≤ 30 year**	**6.52 ± 2.4**	**(1.6,11.3)**	**.008**
**30-40 year**	**4.42 ± 2.2**	**(−.0,8.8)**	**.051**
**> 40 year**	**Reference.**		

^a^*B ± SE* Standard error for the unstandardized beta, *CI* confidence interval

The effects of 3-month endometriosis treatments on QOL and VAS score levels.

In this study, 47.1% (*n* = 48) of the endometriotic women without infertility (group D) were treated with the medical, in 52.9% (*n* = 54) of whom surgical treatments were performed. In the reassessment of the quality of life after 3 months through the same EHP30 questionnaire, the results showed that their total quality of life improved after the treatment (mean 42.3 out of 55.3 for the medical treatment and mean 36.1 out of 60.6 for the surgical treatment). The quality of life also seemed to have grown in the surgical treatment, which was more evident than that in the medical treatment. In terms of examining different parts of this questionnaire, the differences between pain (*P* = 0.002) and social support (*P* = 0.008) before and after the treatments were more noticeable than those among other items. However, there was a significant difference in terms of the reported feeling of well-being (*P* = 0.01) before and after the treatment, but about control and powerlessness (*P* = 0.07) and self-image (*P* = 0.09), the differences were not significant (Table 5).

**Table 5:** Quality of life in the endometriotic patients without infertility via EHP30 questionnaire before and after the treatments

Variable	Type of treatment	Medical group *N* = 50	Surgical group *N* = 50	P1	P2	P3
Pain	Before	54.54 ± 18.04	62.22 ± 22.78	< 0.001	< 0.001	0.002
	After	6.29 ± 3.16	5.15 ± 2.73			
	Difference	48.26 ± 16.29	57.07 ± 21.89			
Control and powerlessness	Before	60.07 ± 22.83	64.69 ± 23.29	< 0.001	< 0.001	0.07
	After	15 ± 7.75	13.64 ± 6.92			
	Difference	45.07 ± 19.20	51.05 ± 20.51			
Emotional well-being	Before	57.57 ± 19.65	61.48 ± 23.03	< 0.001	< 0.001	0.01
	After	16.46 ± 7.26	14.2 ± 7.21			
	Difference	41.11 ± 16.86	47.28 ± 20.02			
Social support	Before	56.56 ± 22.72	58.43 ± 23.27	< 0.001	< 0.001	0.008
	After	25.52 ± 10.58	21.02 ± 11.30			
	Difference	31.04 ± 18.65	37.41 ± 20.18			
Self-image	Before	41.80 ± 21.9	48.39 ± 25.35	< 0.001	< 0.001	0.09
	After	22.64 ± 10.27	20.98 ± 11.54			
	Difference	19.17 ± 20.28	27.41 ± 20.74			
Total	Before	55.306 ± 17.917	60.679 ± 19.545	< 0.001	< 0.001	< 0.001
	After	42.347 ± 15.976	36.111 ± 15.918			
	Difference	12.96 ± 14.14	24.57 ± 16.51			

All the results are presented as the mean ± SD

P1: *P*-value refers to comparisons between month 0 and months 3 within the groups (Wilcoxon or paired t test as appropriate)

P2: *P*-value refers to comparison of the mean among the groups at the end of study (Wilcoxon or paired t test as appropriate)

P3: *P*-value refers to comparison of the differences among the groups (Kruskal-Wallis test or ANOVA as appropriated)

For the endometriotic women who received medical or surgical treatments, a VAS questionnaire was filled before and after the intervention according to their reports of symptoms in order to assess their pain. Based on the obtained results, represented in Table 6, the noncyclic pain (*P* = 0.002), dysmenorrhea (*P* = 0.003), and dyspareunia (*P* = 0.001) were significantly different before and after the treatment; there were, however, no significant differences concerning the gastrointestinal symptoms (*P* = 0.051) and urinary symptoms during menopause (*P* = 0.1).

**Table 6:** Endometriosis-related pain and self-rate assessment in endometriotic patients without infertility via VAS score before and after treatment

Variable	Type of treatment	Medical group *N* = 50	Surgical group *N* = 50	P1	P2	P3
Noncyclic pain	Before	6.64 ± 2.49	7.41 ± 1.80	0 < .001	0 < .001	0.002
	After	3.32 ± 3.58	1.75 ± 2.68			
	Difference	3.32 ± 3.03	5.66 ± 2.48			
Dysmenorrhea	Before	7.72 ± 2.17	8.04 ± 2.16	0 < .001	0 < .001	0.003
	After	3.75 ± 2.77	1.97 ± 2.89			
	Difference	3.97 ± 3.08	6.07 ± 3.09			
Deep dyspareunia	Before	6.11 ± 2.58	6.64 ± 2.37	.008	0 < .001	0.001
	After	4.88 ± 2.96	2.14 ± 2.96			
	Difference	1.22 ± 1.73	4.5 ± 1.93			
GI symptom at menses	Before	5.81 ± 2.78	7.63 ± 2.05	0 < .001	0 < .001	0.051
	After	1.93 ± 2.93	1.27 ± 2.18			
	Difference	3.87 ± 2.55	6.36 ± 2.46			
Urinary symptom at menses	Before	5.833 ± 2.483	6.64 ± 2.23	0 < .001	0 < .001	0.1
	After	1.0 ± 2.44	0.286 ± .726			
	Difference	4.83 ± 2.64	6.35 ± 2.097			
Total	Before	6.42 ± 2.51	7.27 ± 2.27	0 < .001	0 < .001	0 < .001
	After	3.0 ± 2.93	1.48 ± 2.71			
	Difference	3.42 ±2.62	5.79 ± 2.83			

All the results are presented as the mean ± SD

P1: *P*-value refers to comparisons between month 0 and months 3 within the groups (Wilcoxon or paired t test as appropriate)

P2: *P*-value refers to comparisons between the groups at the end of the study (Kruskal-Wallis test or ANOVA as appropriated)

P3: *P*-value refers to comparison of the difference among the groups (Kruskal-Wallis test or ANOVA as appropriated)

Association of quality of life (QOL) level with clinical variables in endometriotic patients.

In order to investigate the association between QOL level and the clinical variables in endometriotic patients, linear regression analysis was performed. The univariate logistic regression model revealed a significant association between QOL and the duration of infertility, VAS score of noncyclic pain, dysmenorrhea, VAS score of dysmenorrhea, dysmenorrhea of dyspareunia, irregular bleeding, and infertility (*P* < 0.05). No significant association was found between QOL level and the clinical characteristics, such as age, socio-economic status, feeling tired, and constipation or diarrhea (*P* > 0.05) (Table 7).

**Table 7:** Investigating the impact of effective clinical variables in improving the quality of life in the endometriosis patients

Clinical variables	B ± SE^a^	95%CI	*P*-value	VIF^b^
Duration of Infertility (year)	0.4 ± .19	0.02, 0.76	0.041	1.42
VAS Score Noncyclic pain	0.94 ± .32	0.32, 1.57	0.003	1.76
Dysmenorrhea	10.79 ± 3.77	3.35, 18.23	0.005	4.41
VAS Score Dysmenorrhea	3.03 ± .5	2.05, 4.01	<.001	5.6
VAS Score Deep dyspareunia	0.69 ± .33	0.03, 1.35	0.042	1.9
Feeling tired	4.11 ± 2.28	−0.39, 8.62	0.07	1.4
Constipation or diarrhea	3.9 ± 2.1	−0.23, 8.05	0.06	1.3
Irregular bleeding	7.74 ± 2.65	2.51, 12.97	0.004	1.3
Age	−2.61 ± 1.4	−5.37, 0.16	0.06	1.2
Social Economic status	−3.27 ± 1.78	−6.79, 0.24	0.06	1.1
Endometriosis Group	1.43 ± .64	0.17, 2.69	0.03	2.4

^a^*B ± SE* Standard error for the unstandardized beta, *CI* confidence interval

^b^Variance Inflation Factor

## Discussion

Endometriosis is a complex estrogen-dependent disease that usually affects women of the reproductive age. It is characterized by the presence of stroma or endometrial glands outside the uterus [23]. Normally, women with endometriosis report significant impairments in QOL since pain and other complications negatively affect this factor. Today, there is no gold standard for assessing QOL in women with endometriosis, and there are different instruments with different scoring systems, response formats and conceptual frameworks for measuring QOL in endometriotic women. Nevertheless, the majority of studies in this field have concluded that women with endometriosis have a lower level of QOL than healthy women in several domains of their life [24]. Our study also clearly indicated that the QOL of women with endometriosis was much lower compared to that of the healthy subjects [3].

In previous studies, where a version of the SF-36 was applied, significant differences were observed between women with endometriosis and controls in terms of physical functioning domain of QOL [25, 26]. This is while generic instruments, such as the SF-36, are helpful for different disorders; they do not however correlate well with pain intensity [27]. Moreover, some of the main problems of endometriosis patients, such as infertility, may not be evaluated well with general questionnaires [28]. Thus, the EHP-30-validated questionnaire based on open-ended exploratory interviews with patients could be highly recommended for endometriotic patients [29], which was used in our study as well.

Likewise, Facchin et al. reported that SF-12 women with endometriosis had worse scores than the control group [30], which shows women with endometriosis can experience significant impairment in their daily lives. In various studies, different affected domains by endometriosis have been reported, but physical performance and pain have been shown to be the most important issues that notably affect their lives [31, 32]. In recent research works, no significant association has been found between higher stages of endometriosis and a lower QOL score because it is depended on individual perception of the disease [8, 9, 33, 34].

Another finding in this study was the improvement in the quality of life and reduction in pain 3 months after endometriosis treatment, which is in agreement with previous papers that measured the quality of life after different periods of treatment. Selecting the best treatment option for patients with endometriosis is believed to be a big challenge and depends on certain factors, such as the patient’s age, the severity of the disease and symptoms, preference for future pregnancy [35, 36]. However, the adverse effect of medical and surgical treatments on the mental status of the endometriotic patients cannot be ignored [14]. Medical treatments include hormonal agents and pain-relieving drugs, which are the first-line endometriosis treatments and their long-term use is safe [37]. According to Sansaon. A et al., etonogestrel implants could improve the quality of life and sexual function and reduce pelvic pain [38]. A number of studies have concluded that adding psychological therapy, such as rational/emotive or cognitive behavioral therapy, can be conducive to improving the emotional status of patients with more satisfying treatment results [39]. Our findings also implied that medical treatments can improve the QOL of endometriotic patients, but the results of surgical interventions seemed to be more significant.

Sometimes, in severe cases with intensive symptoms, in whom medical therapy could not be effective, or for women with endometriosis-associated infertility, surgery is the only remaining choice [35]. Moreover, other papers have concluded that patients sometimes seem to have better experiences with surgery treatment; they are those who manifest palpable lesions within the pelvis and have severe pelvic pain [40, 41].

According to the results of a systematic review and meta-analysis study, for all types of endometriosis, surgery enhances the quality of life in the majority of health domains, using either generic or endometriosis-specific QOL questionnaires [42]. According to our results, surgical treatment was effective in improving the QOL and reducing pain sensation. Ballester M et al. measured Bristol Female Lower Urinary Tract Symptoms (BFLUTS) and postoperative quality of life 3 months following the surgery and compared the obtained scores with preoperative ones. They reported a significant improvement in both times (*P* = 0.005 and *P* = 0.001, respectively) [43]. Another study, in which the EHP30 questionnaire was used and the time of follow-up was the same as ours, concluded that bilateral salpingo-oophorectomy and total abdominal hysterectomy significantly improved QOL in endometriotic patients [44]. Meanwhile, in studies conducted for far longer periods, such as that of Porpora et al. with 3 years of follow-up [45] or the study by Abotte et al. with 2 to 5 years of follow-up, similar results were reported concerning the effectiveness of surgical treatment [28]. However, pain assessment in these patients cannot be considered in general as an accurate indicator since pain is multifactorial and painful symptoms do not only arise due to endometriotic lesions but also may be explained by postoperative side effects, complications, disease recurrences, and persistent [40]; for example Aurélie et al., evaluated the long-term quality of life following surgical treatment for endometriosis. A number of patients reported a high quality of life score after surgery even though the pain had not completely disappeared whereas certain reported a poor quality of life despite no longer having pain [46].

There is some strength in the present work, which could be highlighted; for instance, only the patients who had a confirmed diagnosis of endometriosis were included in the study, instead of those with only suggestive symptoms. Moreover, different QOL questionnaires were used herein. This study examined the quality of life in endometriosis patients with and without infertility and compare the effects of surgical or medical treatment on the quality of life of these patients.

On the other hand, this work had a number of limitations; to begin with, it was conducted in a medical center specialized in infertility issues, as a result of which the patients may have particularly severe forms of infertility problems or severe endometriosis. Secondly, the diagnosis of endometriosis in our study was confirmed by the presence of clinical symptoms associated with endometriosis signs, using ultrasound findings. Hence, it should be mentioned that some women with asymptomatic superficial endometriosis were erroneously included in the none-endometriosis group.

## Conclusion

Endometriosis reduces the QOL and if infertility is added to it, this negative effect even further exacerbates. However, surgical or medical treatment improves the QOL and choosing the best strategy out of these two methods, depends on the patient’s conditions.

## Data Availability

All respectable readers and researchers can request the data by directly contacting the primary author at ta.poordast@yahoo.com

## Abbreviations

QOL: Quality of life
EHP-30: Endometriosis Health Profile-30
WES: World Endometriosis Societies
OCP: Oral contraceptive
NSAID: Non-steroidal anti-inflammatory drugs
VAS: Visual analog scale
CPP: Chronic pelvic pain
OLS: Ordinary least squared

## References

[1] Sampson JA (1927). Metastatic or embolic endometriosis, due to the menstrual dissemination of endometrial tissue into the venous circulation. Am J Pathol.

[2] Ozkan S, Murk W, Arici A (2008). Endometriosis and infertility: epidemiology and evidence-based treatments. Ann N Y Acad Sci.

[3] Wu MH, Su PF, Chu WY, Lin CW, Huey NG, Lin CY, Ou HT. Quality of life among infertile women with endometriosis undergoing IVF treatment and their pregnancy outcomes. J Psychosom Obstet Gynaecol. 2021;42(1):57-66. 10.1080/0167482X.2020.1758659 32345090

[4] Adamson GD, Kennedy S, Hummelshoj L (2010). Creating solutions in endometriosis: global collaboration through the world endometriosis Research Foundation. In., vol. 2.

[5] Jones G, Jenkinson C, Kennedy S (2004). The impact of endometriosis upon quality of life: a qualitative analysis. J Psychosom Obstet Gynaecol.

[6] Kennedy S, Bergqvist A, Chapron C, D'Hooghe T, Dunselman G, Greb R (2005). ESHRE guideline for the diagnosis and treatment of endometriosis. Human Reprod (Oxford, England).

[7] Vercellini P, Frattaruolo MP, Somigliana E, Jones GL, Consonni D, Alberico D (2013). Surgical versus low-dose progestin treatment for endometriosis-associated severe deep dyspareunia II: effect on sexual functioning, psychological status and health-related quality of life. Human Reprod (Oxford, England).

[8] Laganà AS, La Rosa VL, Rapisarda AMC, Valenti G, Sapia F, Chiofalo B (2017). Anxiety and depression in patients with endometriosis: impact and management challenges. Int J Women's Health.

[9] Vitale SG, Petrosino B, La Rosa VL, Rapisarda AM, Laganà AS (2016). A systematic review of the association between psychiatric disturbances and endometriosis. J Obstetr Gynaecol Canada: JOGC =Journal d'obstetrique et gynecologie du Canada : JOGC.

[10] Mignemi G, Facchini C, Raimondo D, Montanari G, Ferrini G, Seracchioli R (2012). A case report of nasal endometriosis in a patient affected by Behcet's disease. J Minim Invasive Gynecol.

[11] Laganà AS, Condemi I, Retto G, Muscatello MR, Bruno A, Zoccali RA (2015). Analysis of psychopathological comorbidity behind the common symptoms and signs of endometriosis. Eur J Obstet Gynecol Reprod Biol.

[12] Minson FP, Abrão MS, Sardá Júnior J, Kraychete DC, Podgaec S, Assis FD (2012). Importance of quality of life assessment in patients with endometriosis. Revista brasileira de ginecologia e obstetricia : revista da Federacao Brasileira das Sociedades de Ginecologia e Obstetricia.

[13] D’Alterio MN, Saponara S, Agus M, Laganà AS, Noventa M, Loi ES (2021). Medical and surgical interventions to improve the quality of life for endometriosis patients: a systematic review. Gynecol Surg.

[14] Vitale SG, La Rosa VL, Rapisarda AMC, Laganà AS. Impact of endometriosis on quality of life and psychological well-being. J Psychosom Obstet Gynaecol. 2017;38(4):317-9. 10.1080/0167482X.2016.1244185. Epub 2016 Oct 18. 10.1080/0167482X.2016.1244185 27750472

[15] Raimondo D, Mabrouk M, Zannoni L, Arena A, Zanello M, Benfenati A (2018). Severe ureteral endometriosis: frequency and risk factors. J Obstetr Gynaecol.

[16] Seracchioli R, Ferrini G, Montanari G, Raimondo D, Spagnolo E, Di Donato N (2015). Does laparoscopic shaving for deep infiltrating endometriosis alter intestinal function? A prospective study. Aust N Z J Obstet Gynaecol.

[17] Spagnolo E, Zannoni L, Raimondo D, Ferrini G, Mabrouk M, Benfenati A (2014). Urodynamic evaluation and anorectal manometry pre- and post-operative bowel shaving surgical procedure for posterior deep infiltrating endometriosis: a pilot study. J Minim Invasive Gynecol.

[18] Johnson NP, Hummelshoj L (2013). Consensus on current management of endometriosis. Human Reprod (Oxford, England).

[19] Adamson GD, Nelson HP (1997). Surgical treatment of endometriosis. Obstet Gynecol Clin N Am.

[20] Wickström K, Edelstam G (2017). Minimal clinically important difference for pain on the VAS scale and the relation to quality of life in women with endometriosis. Sex Reprod Healthcare.

[21] Abulnaja KO, El Rabey HA (2015). The efficiency of barley (Hordeum vulgare) bran in ameliorating blood and treating fatty heart and liver of male rats. Evid-based Complement Altern Med.

[22] Boskabady MH, Kiani S, Rakhshandah H (2006). Relaxant effects of Rosa damascena on Guinea pig tracheal chains and its possible mechanism(s). J Ethnopharmacol.

[23] Vercellini P, Viganò P, Somigliana E, Fedele L (2014). Endometriosis: pathogenesis and treatment. Nat Rev Endocrinol.

[24] Jia SZ, Leng JH, Shi JH, Sun PR, Lang JH (2012). Health-related quality of life in women with endometriosis: a systematic review. J Ovarian Res.

[25] Lövkvist L, Boström P, Edlund M, Olovsson M (2016). Age-related differences in quality of life in Swedish women with endometriosis. J Women's Health (2002).

[26] Nunes FR, Ferreira JM, Bahamondes L (2014). Prevalence of fibromyalgia and quality of life in women with and without endometriosis. Gynecol Endocrinol.

[27] Marques A, Bahamondes L, Aldrighi JM, Petta CA (2004). Quality of life in Brazilian women with endometriosis assessed through a medical outcome questionnaire. J Reprod Med.

[28] Abbott JA, Hawe J, Clayton RD, Garry R (2003). The effects and effectiveness of laparoscopic excision of endometriosis: a prospective study with 2-5 year follow-up. Human Reprod (Oxford, England).

[29] Jones G, Kennedy S, Barnard A, Wong J, Jenkinson C (2001). Development of an endometriosis quality-of-life instrument: the endometriosis health Profile-30. Obstet Gynecol.

[30] Facchin F, Barbara G, Saita E, Mosconi P, Roberto A, Fedele L (2015). Impact of endometriosis on quality of life and mental health: pelvic pain makes the difference. J Psychosom Obstet Gynaecol.

[31] De Graaff AA, D'Hooghe TM, Dunselman GA, Dirksen CD, Hummelshoj L, Simoens S (2013). The significant effect of endometriosis on physical, mental and social wellbeing: results from an international cross-sectional survey. Human Reprod (Oxford, England).

[32] Fourquet J, Báez L, Figueroa M, Iriarte RI, Flores I (2011). Quantification of the impact of endometriosis symptoms on health-related quality of life and work productivity. Fertil Steril.

[33] Casu G, Ulivi G, Zaia V, Fernandes Martins MDC, Parente Barbosa C, Gremigni P. Spirituality, infertility-related stress, and quality of life in Brazilian infertile couples: Analysis using the actor-partner interdependence mediation model. Res Nurs Health. 2018;41(2):156-65. 10.1002/nur.21860. Epub 2018 Feb 5. 10.1002/nur.21860 29399819

[34] Pessoa de Farias Rodrigues M, Lima Vilarino F, de Souza Barbeiro Munhoz A, da Silva Paiva L, de Alcantara Sousa LV, Zaia V, Parente Barbosa C. Clinical aspects and the quality of life among women with endometriosis and infertility: a cross-sectional study. BMC Womens Health. 2020;20(1):124. 10.1186/s12905-020-00987-7. 10.1186/s12905-020-00987-7 PMC7291762 32532273

[35] de Paula AM, Borrelli GM, Kho RM, Abrão MS (2017). The current management of deep endometriosis: a systematic review. Minerva Ginecol.

[36] Kho RM, Andres MP, Borrelli GM, Neto JS, Zanluchi A, Abrão MS (2018). Surgical treatment of different types of endometriosis: comparison of major society guidelines and preferred clinical algorithms. Best Pract Res Clin Obstetr Gynaecol.

[37] Berlanda N, Somigliana E, Viganò P, Vercellini P (2016). Safety of medical treatments for endometriosis. Expert Opin Drug Saf.

[38] Sansone A, De Rosa N, Giampaolino P, Guida M, Laganà AS, Di Carlo C. Effects of etonogestrel implant on quality of life, sexual function, and pelvic pain in women suffering from endometriosis: results from a multicenter, prospective, observational study. Arch Gynecol Obstet. 2018;298(4):731-6. 10.1007/s00404-018-4851-0. Epub 2018 Aug 3. 10.1007/s00404-018-4851-0 30074068

[39] González-Echevarría AM, Rosario E, Acevedo S, Flores I. Impact of coping strategies on quality of life of adolescents and young women with endometriosis. J Psychosom Obstet Gynaecol. 2019;40(2):138-45. 10.1080/0167482X.2018.1450384. Epub 2018 Apr 12. 10.1080/0167482X.2018.1450384 PMC6185815 29648907

[40] Comptour A, Pereira B, Lambert C, Chauvet P, Grémeau AS, Pouly JL (2020). Identification of predictive factors in endometriosis for improvement in patient quality of life. J Minim Invasive Gynecol.

[41] Healey M, Ang WC, Cheng C (2010). Surgical treatment of endometriosis: a prospective randomized double-blinded trial comparing excision and ablation. Fertil Steril.

[42] Arcoverde FVL, Andres MP, Borrelli GM, Barbosa PA, Abrão MS, Kho RM (2019). Surgery for endometriosis improves major domains of quality of life: a systematic review and meta-analysis. J Minim Invasive Gynecol.

[43] Ballester M, Dubernard G, Wafo E, Bellon L, Amarenco G, Belghiti J (2014). Evaluation of urinary dysfunction by urodynamic tests, electromyography and quality of life questionnaire before and after surgery for deep infiltrating endometriosis. Eur J Obstet Gynecol Reprod Biol.

[44] Tan BK, Maillou K, Mathur RS, Prentice A (2013). A retrospective review of patient-reported outcomes on the impact on quality of life in patients undergoing total abdominal hysterectomy and bilateral salpingo-oophorectomy for endometriosis. Eur J Obstet Gynecol Reprod Biol.

[45] Porpora MG, Pallante D, Ferro A, Crisafi B, Bellati F, Benedetti Panici P (2010). Pain and ovarian endometrioma recurrence after laparoscopic treatment of endometriosis: a long-term prospective study. Fertil Steril.

[46] Comptour A, Lambert C, Chauvet P, Figuier C, Gremeau AS, Canis M, Pereira B, Bourdel N. Long-Term Evolution of Quality of Life and Symptoms Following Surgical Treatment for Endometriosis: Different Trajectories for Which Patients? J Clin Med. 2020;31;9(8):2461. 10.3390/jcm9082461 PMC7463511 32752110

